# Serum Fructosamine Concentration in Uncontrolled Hyperthyroid Diabetic Cats Is within the Population Reference Interval

**DOI:** 10.3390/vetsci4010017

**Published:** 2017-03-15

**Authors:** Arnon Gal, Brie Trusiano, Adrienne F. French, Nicolas Lopez-Villalobos, Amy L. MacNeill

**Affiliations:** 1 Institute of Veterinary, Animals and Biomedical Sciences, Massey University, Tennent Drive, Palmerston North 4442, New Zealand; N.Lopez-Villalobos@massey.ac.nz; 2 Microbiology, Immunology & Pathology Dept., Colorado State University, 1682 Campus Delivery, Fort Collins, CO 80523, USA; brietrusiano1@gmail.com (B.T.); amy.macneill@colostate.edu (A.L.M.); 3 New Zealand Veterinary Pathology/IDEXX, Tennent Drive, Palmerston North 4442, New Zealand; a.french@massey.ac.nz

**Keywords:** diabetes mellitus, felis catus, fructosamine, hyperthyroidism

## Abstract

Diabetes mellitus is a common endocrinopathy of cats that is characterized by persistent fasting hyperglycemia. However, stress induces substantial hyperglycemia in cats that poses a challenge to the veterinarian who may wrongly interpret the high serum concentration of blood glucose as evidence of diabetes mellitus. Fructosamine is a glycated serum protein that serves as an index of glycemic control in cats and is useful because it is not affected by stress hyperglycemia. However, factors such as body weight, hypoproteinemia, and increased serum thyroid hormone concentration can alter fructosamine concentration. The goal of this retrospective study was to compare the fructosamine concentrations in diabetic and nondiabetic cats with and without uncontrolled hyperthyroidism. A secondary goal was to determine the effect of sex, age, different populations of cats, and diabetes on the variability of fructosamine. We found that the mean (±SE) serum fructosamine of hyperthyroid diabetic cats (332 ± 24 µmol/L, 95% CI 291–379 µmol/L) was within the population-based reference interval (200–360 µmol/L) and significantly lower in comparison to euthyroid diabetic cats (527 ± 10 µmol/L, 95% CI 515–553 µmol/L). Additionally, in this study, diabetes accounted only for approximately 50% of the variance in serum fructosamine, while age, sex, and population made a minor contribution to this variance. In conclusion, finding serum fructosamine that is within the population-based reference interval in an uncontrolled diabetic cat should alert the veterinarian to the possibility of concurrent hyperthyroidism. Additionally, the veterinary clinician should consider that serum fructosamine might be substantially affected by factors other than diabetes.

## 1. Introduction

Diabetes mellitus (DM) is a common feline endocrinopathy. A recent cross-sectional study from the United Kingdom reported a prevalence of 0.58% for DM in a cohort of 193,563 cats from primary-care practices [1]. The hallmark clinicopathologic abnormality of DM is persistent fasting hyperglycemia [2]. However, in cats, stress induces substantial hyperglycemia [3] and poses a challenge to the veterinarian who may consider a high blood glucose concentration evidence of DM in a nondiabetic cat [4]. Fructosamine is a serum glycated protein that serves as an index of glycemic control in diabetic cats [5,6,7,8], and is not affected by stress hyperglycemia [5].

Several factors affect serum fructosamine concentration ([fructosamine]) in cats. The mean [fructosamine] is greater in cats with a body weight of more than 4 kg than in cats with a body weight of less than 4 kg, and mean body weight-adjusted [fructosamine] in cats is greater in males than in females [9]. Hypoproteinemia and hyperthyroidism also affect [fructosamine] by decreasing it [10,11]. 

Hyperthyroidism is the most common endocrinopathy of cats with a reported prevalence of 4%–11% [12,13,14,15,16]. Despite the high prevalence of hyperthyroidism and DM as separate entities, the concomitance of both diseases is uncommon [17,18]. Furthermore, the clinical utility of [fructosamine] in the hyperthyroid diabetic cat is unknown, and it has been suggested that the hyperthyroid state would lead to decreased [fructosamine] in diabetics [10]. Therefore, the goal of this study was to compare [fructosamine] in diabetic and nondiabetic cats with and without uncontrolled hyperthyroidism. We hypothesized that [fructosamine] in hyperthyroid diabetic cats would be lower than in euthyroid diabetic cats. In addition, we wanted to estimate the variance components for the different sources of variation that affect [fructosamine] in two large cohorts of diabetic cats in New Zealand and Colorado.

## 2. Materials and Methods

### 2.1. Databases

The medical records of New Zealand Veterinary Pathology (NZVP) and The Colorado State University Veterinary Teaching Hospital (CSU-VTH) were retrospectively searched between years 2004–2015 and 2000–2015, respectively. Cats were included in the study if their medical records had results of serum total thyroxine (TT4) and [fructosamine] from the same visit. Additional information that was recorded included signalment, serum glucose and the final diagnoses when those were available.

In the NZVP cohort, the authors made the diagnosis of DM when the referring veterinarian identified the animal as a diabetic or indicated that the cat is treated with insulin on the submission form, or if serum glucose concentration (when available) was >19.5 mmol/L (351 mg/dL) [3]. Cases for which glucose and history were not available were excluded from any analysis in which DM was an independent variable. According to these criteria, 90 cats had the diagnosis of DM based on history and 114 cats had the diagnosis of DM based on serum glucose >19.5 mmol/L. In the CSU-VTH cohort, the authors made the diagnosis of DM when it appeared in the final diagnostic code in the medical records.

The diagnosis of uncontrolled hyperthyroidism was made when TT4 concentration was above the reference interval (20–40 nmol/L). The reference interval for serum fructosamine was 190–365 µmol/L [6].

### 2.2. Statistical Analysis

All statistical analyses were performed with the Statistical Analysis System software, version 9.3 (SAS Institute Inc., Cary, NC, USA, 2012).

The MIXED procedure was used to determine the effect of DM and uncontrolled hyperthyroidism on [fructosamine] with a linear model that included the fixed effects of population, DM, uncontrolled hyperthyroidism and the interaction between DM and uncontrolled hyperthyroidism. Least squares means and standard errors of [fructosamine] for combinations of DM and uncontrolled hyperthyroidism within each population were obtained and used to perform multiple comparisons.

Estimates of regression coefficients of the regression lines of [fructosamine] and glucose on TT4 were obtained using the GLM procedure. Values of TT4 were normalized using the natural logarithm transformation.

Only data from the NZVP cohort was included in the comparison of [fructosamine] between euthyroid and hyperthyroid nondiabetic and diabetic cats. This was because the CSU-VTH cohort did not have any case for which [fructosamine] and TT4 were analyzed from the same sample. Similarly, the estimates of the regression lines of [fructosamine] and glucose on TT4 were performed only on the NZVP cohort.

Estimates of variance components for the different sources of variation affecting [fructosamine] were obtained using the MIXED procedure with a model that included the random effects of population, DM, age, sex and residual. Serum total protein was not used as a covariate in this analysis because none of the cats were hypoproteinemic.

## 3. Results

The descriptive and demographic data for the NZVP and CSU-VTH are presented in Table 1.

**Figure 1:**
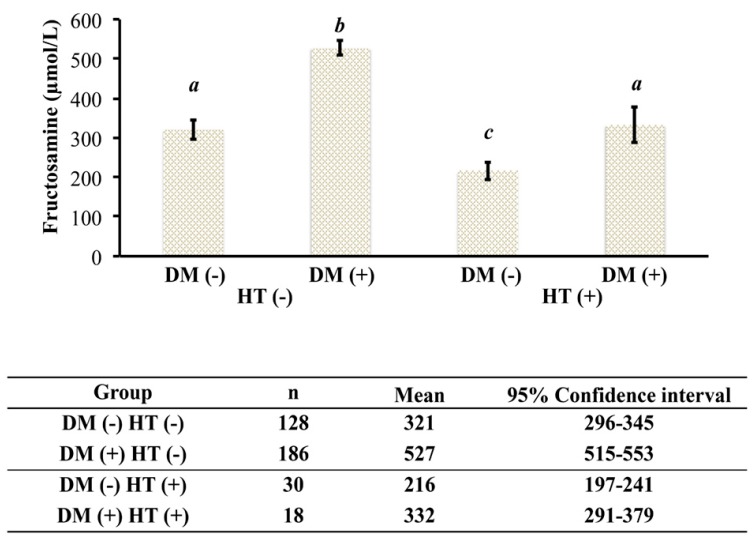
[Fructosamine] in euthyroid and hyperthyroid nondiabetic and diabetic cats from New Zealand. DM (−), nondiabetic; DM (+), diabetic; HT (−), euthyroid; HT (+), hyperthyroid. Groups with different letters are statistically significantly different (*p* < 0.01).

The NZVP cohort was divided into four groups: (1) sick euthyroid nondiabetic; (2) euthyroid diabetic; (3) hyperthyroid nondiabetic; and (4) hyperthyroid diabetic. First, we calculated the prevalence of concurrent uncontrolled hyperthyroidism and DM and found it was 5%. Further, we found that the mean (±SE) [fructosamine] of hyperthyroid diabetic cats (332 ± 24 µmol/L, 95% CI 291–379 µmol/L; *n* = 18) was within the population reference interval (200–360 µmol/L). Additionally, mean (±SE) [fructosamine] in hyperthyroid diabetic cats was similar to the mean (±SE) [fructosamine] of sick euthyroid nondiabetic cats (321 ± 13 µmol/L, 95% CI 296–345 µmol/L; *n* = 128). In contrast, there was a significant difference in [fructosamine] means (±SE) between sick euthyroid nondiabetic cats (321 + 13 µmol/L, 95% CI 296–345 µmol/L; *n* = 128) and hyperthyroid nondiabetic cats (216 ± 13 µmol/L, 95% CI 197–241 µmol/L; *n* = 30; *p* < 0.05). Finally, there was a significant difference between the means (±SE) of [fructosamine] of euthyroid diabetic cats (527 ± 10 µmol/L, 95% CI 515–553 µmol/L; *n* = 186) and hyperthyroid diabetic cats (332 ± 24 µmol/L, 95% CI 291–379 µmol/L; *n* = 18; *p* < 0.05; Figure 1).

**Figure 2:**
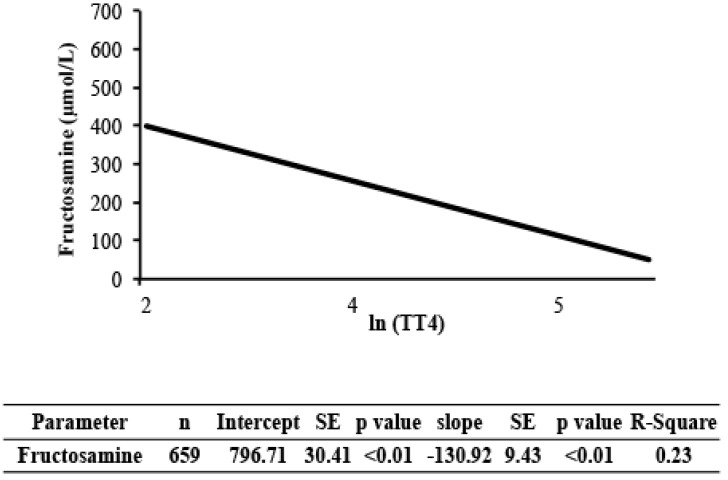
Regression analysis of [fructosamine] on total thyroxine (TT4) in cats from New Zealand. Values of TT4 were normalized using the natural logarithm transformation. TT4, serum total thyroxine concentration; SE, standard error; R-square, coefficient of determination.

**Figure 3:**
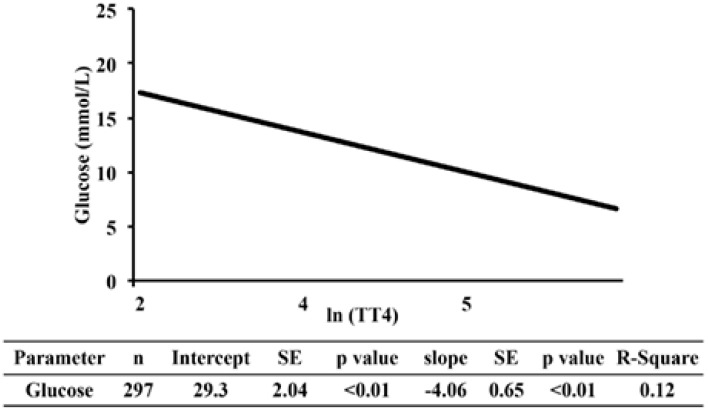
Regression analysis of serum glucose on TT4 in cats from New Zealand. Values of TT4 were normalized using the natural logarithm transformation. TT4, serum total thyroxine concentration; SE, standard error; R-square, coefficient of determination.

Estimates of the regression lines of [fructosamine] and glucose on TT4 for the NZVP cohort are presented in Figure 2 and Figure 3, respectively. The slopes were negative and significant meaning that as the concentration of TT4 increases there is a reduction in [fructosamine] and glucose.

**Figure 4:**
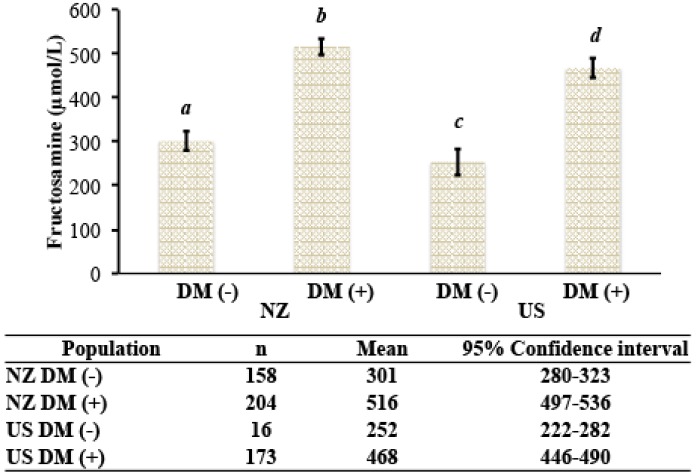
[Fructosamine] in nondiabteic and diabetic cats from New Zealand and the United States. DM (−), nondiabetic; DM (+), diabetic; NZ, New Zealand; U.S., United States. Groups with different letters are statistically significantly different (*p* < 0.01).

Last, we performed the analysis of variance components for the different sources of variation affecting [fructosamine]. In both cohorts, diabetic cats had significantly higher [fructosamine] than nondiabetic cats (Figure 4). The estimates of the variance components that explain the variation in [fructosamine] are presented in Table 2. Diabetes mellitus was the main factor that explained the variation of [fructosamine] (49.65%), while age, sex, and population made minor contributions.

## 4. Discussion

In this study, the authors report a low prevalence of concurrent uncontrolled hyperthyroidism and DM (18/362; 5%) that is remarkably similar to that described in two previous reports [17,18]. The study also confirmed an earlier concern [6] that [fructosamine] in hyperthyroid diabetics might be lower than that of the euthyroid diabetic cats (Figure 1). Moreover, the results indicated that [fructosamine] was within the population-based reference interval in hyperthyroid diabetic cats and not different than that of the sick euthyroid nondiabetic cats. This finding has a significant clinical implication when a [fructosamine] within the reference interval in a diabetic cat with uncontrolled hyperthyroidism is wrongly interpreted by the clinician as consistent with insulin overdose and forthcoming diabetic remission [19,20,21]. Therefore, the authors conclude that there is no clinical utility for measuring [fructosamine] in a diabetic cat with confirmed uncontrolled hyperthyroidism, and recommend testing for hyperthyroidism in the clinically uncontrolled diabetic cat that has [fructosamine] within the population-based reference interval.

This study also found a significant, weak negative association between TT4 and glucose concentrations. One explanation for the negative association between TT4 and glucose concentrations is that hyperthyroidism stimulates the uptake and utilization of glucose. A recent clinical trial in people is in support of this argument. In that study [22], oral supplementation of levothyroxine increased thyroid hormone levels in both treated hypothyroid human patients and healthy volunteers who become hyperthyroid. In both groups, there was a thyroxine-dependent increased glucose disposal in tissues and an improved, rather than deteriorated, index of insulin action. Moreover, hyperglycemia is uncommon in feline hyperthyroidism, with only 3% of hyperthyroid cats developing hyperglycemia [23]. Conversely, a possible reason why hyperthyroid cats do not commonly develop hypoglycemia might be because of a concurrent increase in the activity of the Cori cycle, and subsequently gluconeogenesis, secondary to the effect of thyroid hormones [24].

A counter argument for the negative correlation between TT4 and glucose concentrations in our study would be that systemic disease possibly led to a decrease in TT4 concentration and an increase in serum glucose concentration (i.e., euthyroid sick syndrome [25] and insulin antagonism secondary to systemic inflammation). Because of the retrospective study design, we could not determine the cause and effect relationship between TT4 and glucose concentrations. To determine the causality between TT4 and glucose, one will need, for example, to discontinue methimazole treatment in clinically-controlled hyperthyroid cats and medicate euthyroid cats with levothyroxine.

The variance component analysis indicated that factors not included in the model (residual variance) were responsible for approximately 45% of the variance in [fructosamine]. The effects of age, sex, and population made minor contributions to this variance. One factor that was previously shown to correlate weakly with [fructosamine] is body weight [9]. Body weight was not recorded on the laboratory submission forms and hence could not be evaluated. Therefore its effect on the variance of [fructosamine] in this study is unknown. Two other known factors that affect [fructosamine] include hyperthyroidism and hypoproteinemia [6,10]. The cats in this study were not hypoproteinemic. Furthermore, we could not analyze the hyperthyroid status in the variance component analysis because the Colorado State University cohort did not have samples with the concurrent submission of serum for analysis of [fructosamine] and TT4. While one could consider that uncontrolled hyperthyroidism affected the variance component analysis, we do not expect that it was responsible for 45% of the change in the variance of [fructosamine]. This assumption is supported by our finding that TT4 concentrations were weakly associated with [fructosamine] (*r* = 0.5; Figure 2). Hence, we conclude that there are additional, yet unknown factors that substantially alter [fructosamine] in cats.

This study had several limitations derived from its retrospective nature. First, in cats for which anamnestic information was not available on the admission form, the authors based the diagnosis of DM on glucose levels that exceed an arbitrary cut-off. The authors established this cut-off value based on a study in which stress induced hyperglycemia in a controlled experimental setting [3]. Second, only cats with TT4 concentration above the laboratory reference range were considered hyperthyroid. The reader should recognize that some of the cats with TT4 concentration within the upper reference range could have been hyperthyroid (i.e., “occult hyperthyroidism”) [23,26]. Third, the euthyroid nondiabetic cats cannot be regarded as healthy controls, and in many the underlying disease was unknown. Therefore, we have no way to determine if their underlying conditions had an effect on glucose, [fructosamine], and TT4 concentrations. Fourth, the duration of sickness was largely unknown in most cats, and there is the potential that in some, there was not enough time for alterations in [fructosamine] to occur [27]. Fifth, the neuter status was not reported for the majority of cats from the New Zealand cohort and consequently, neuter status was not included in any of the analyses. Lastly, the Colorado State University cohort did not have samples in which [fructosamine] and TT4 concentrations were measured simultaneously. Hence, we could not perform the same analysis on [fructosamine] and TT4 concentrations in that cohort as we did in the New Zealand cohort. Nevertheless, the large number of cats included in the study conferred an advantage, as it allowed us to overcome some of the above limitations that are common to retrospective studies.

## 5. Conclusions

This study indicates that there is no clinical utility for measuring [fructosamine] in a diabetic cat with confirmed uncontrolled hyperthyroidism. Finding [fructosamine] that is within the population-based reference interval in an uncontrolled diabetic cat should alert the veterinarian for the possibility of concurrent hyperthyroidism. Additionally, the veterinarian should consider that [fructosamine] might be substantially affected by factors other than DM.

## Figures and Tables

**Table 1:** Descriptive and demographic data for the New Zealand Veterinary Pathology (NZVP) and Colorado State University Veterinary Teaching Hospital (CSU-VTH) cohorts.

Parameter	NZVP	CSU-VTH
*n*	659	189
Age (years; mean ± SD; min–max)	11 ± 4; 1–24	13 ± 5; 1–26
Males	281	61
Females	343	126
Unknown sex	35	2
Fructosamine (mean ± SD) µmol/L	384 ± 182 (*n* = 659)	449 ± 154 (*n* = 187)
TT4 (mean ± SD) nmol/L	31 ± 33 (*n* = 659)	N/A
Glucose (mean ± SD) mmol/L	16.8 ± 7.9 (*n* = 297)	N/A
Domestic Shorthair	416	131
Domestic Longhair	66	41
Burmese	45	0
Domestic Mediumhair	18	0
Birman	8	0
Siamese	6	8
Ragdoll	4	0
Abyssinian	3	1
Tonkinese	3	0
Maine Coon	2	2
Russian Blue	2	0
Persian cross	2	0
Balinese	1	0
Birman	1	0
British	1	0
British Blue	1	0
Burmese cross	1	0
Chinchilla	1	0
Cornish Rex	1	0
Manx	1	0
Ocicat	1	0
Oriental cross	1	1
Persian	1	0
Ragdoll cross	1	0
Siamese cross	1	0
Somali	1	0
Sphinx	1	0
Himalayan	0	1
unknown	69	4

**Table 2:** Estimates of variance components that explain the variation in [fructosamine] in cats.

Parameter	Effect (%)
Age	3.13
Sex	0.00
Population	1.81
Diabetes	49.65
Residual	45.40
